# Tasimelteon safely and effectively improves sleep in Smith–Magenis syndrome: a double-blind randomized trial followed by an open-label extension

**DOI:** 10.1038/s41436-021-01282-y

**Published:** 2021-07-27

**Authors:** Christos M. Polymeropoulos, Justin Brooks, Emily L. Czeisler, Michaela A. Fisher, Mary M. Gibson, Kailey Kite, Sandra P. Smieszek, Changfu Xiao, Sarah H. Elsea, Gunther Birznieks, Mihael H. Polymeropoulos

**Affiliations:** 1 Vanda Pharmaceuticals Inc., Washington, DC USA; 2 Department of Molecular and Human Genetics, Baylor College of Medicine, Houston, TX USA

## Abstract

**Purpose:**

To assess the efficacy of tasimelteon to improve sleep in Smith–Magenis syndrome (SMS).

**Methods:**

A 9-week, double-blind, randomized, two-period crossover study was conducted at four US clinical centers. Genetically confirmed patients with SMS, aged 3 to 39, with sleep complaints participated in the study. Patients were assigned to treatment with tasimelteon or placebo in a 4-week crossover study with a 1-week washout between treatments. Eligible patients participated in an open-label study and were followed for >3 months.

**Results:**

Improvement of sleep quality (DDSQ50) and total sleep time (DDTST50) on the worst 50% of nights were primary endpoints. Secondary measures included actigraphy and behavioral parameters. Over three years, 52 patients were screened, and 25 patients completed the randomized portion of the study. DDSQ50 significantly improved over placebo (0.4, *p* = 0.0139), and DDTST50 also improved (18.5 minutes, *p* = 0.0556). Average sleep quality (0.3, *p* = 0.0155) and actigraphy-based total sleep time (21.1 minutes, *p* = 0.0134) improved significantly, consistent with the primary outcomes. Patients treated for ≥90 days in the open-label study showed persistent efficacy. Adverse events were similar between placebo and tasimelteon.

**Conclusion:**

Tasimelteon safely and effectively improved sleep in SMS.

## INTRODUCTION

Smith–Magenis syndrome (SMS; OMIM 182290) is a rare genetic disorder that results from an interstitial deletion of 17p11.2 and, in rare cases, from a retinoic acid induced 1 (*RAI1*) gene variant [1]. The prevalence is estimated to be 1/15,000–25,000 [2, 3]. Recently, advancements in genetic testing and educational awareness of SMS have led to increased diagnosis of the syndrome among patients with neurodevelopment deficits [3]. Individuals with SMS present with a distinct pattern of mild to moderate intellectual disability, delayed speech and language skills, distinctive craniofacial and skeletal abnormalities, behavioral disturbances, and, almost uniformly, significant sleep disturbances [4]. Currently, the prevailing theory is that there is an underlying circadian pathophysiology causing sleep disturbances in these patients, as they exhibit low overall melatonin concentrations and abnormal timing of peak plasma melatonin concentrations. This abnormal inverted circadian rhythm is estimated to occur in 95% of individuals with SMS [5, 6].

*RAI1* is a dosage-sensitive gene expressed in many tissues and highly conserved among species. Many studies have demonstrated that RAI1 and its homologs act as a transcriptional factor implicated in embryonic neurodevelopment and neuronal differentiation, as well as behavioral functions and circadian activity. Patients with *RAI1* pathogenic variants show some phenotypic differences when compared to those carrying the typical deletion [7]; however, haploinsufficiency of *RAI1* is the primary cause of the neurobehavioral and metabolic phenotype in SMS.

The 17p11.2 deletion encompasses *RAI1*, leading to haploinsufficiency, which is considered the primary cause for most features of SMS, including dysregulation of the molecular clock via its effect on *CLOCK* expression. ChIP-Chip and reporter studies suggest that RAI1 binds, directly or in a complex, to the first intron of *CLOCK*, enhancing transcriptional activity; thus, reduced expression of *RAI1* results in reduced *CLOCK* expression in both animal models and SMS patient-derived cells [8]. These data suggest that treatment with a circadian regulator can, in part, correct the deficiencies caused by *RAI1* abnormalities, providing further evidence of RAI1 interaction with the molecular clock and the impact on circadian rhythm.

The severe sleep disorder seen in this population causes significant disruption in the lives of individuals with SMS and their families. Sleep for these patients is characterized by difficulty sleeping at night and resultant excessive daytime sleepiness [9–13]. Individuals with SMS have decreased total night sleep, lower sleep efficiency, earlier sleep onset, final sleep offset, and increased waking after sleep onset compared to healthy individuals of the same age [14]. During these nighttime awakenings, individuals with SMS can pose a significant danger to themselves and disrupt the sleep of their family members. Such challenges that families of individuals with SMS face are well documented, and discussed by the advocacy group Parents & Researchers Interested in Smith–Magenis Syndrome (PRISMS) in their Medical Management Guidelines for an Individual Diagnosed with SMS [15].

Prior to the approval of tasimelteon for the treatment of nighttime sleep disturbances in Smith–Magenis Syndrome, there were no FDA-approved treatments for the disrupted sleep patterns associated with SMS. Multiple unapproved treatments are used with limited efficacy and with significant side effects, including hypnotic drugs, antidepressants, antipsychotics, sleep aids, mood stabilizers, α-2 agonists, and benzodiazepines [16]. Previous treatments for the sleep disorder in SMS include melatonin, oral β-1-adrenergic antagonists, and acebutolol with melatonin [17]. The support for this treatment regimen comes from early anecdotal reports and uncontrolled studies. In addition to medications, other methods caregivers use for managing SMS-related behaviors include using locks on doors and safety sleepers.

Tasimelteon is a melatonin receptor agonist that demonstrates high affinity for both the human melatonin MT_1_ and MT_2_ receptors. By acting upon the MT_1_ and MT_2_ receptors, tasimelteon acts by entraining circadian sleep phase timing and has been shown to improve nighttime sleep as well as daytime sleepiness and functioning [18–20]. Tasimelteon is approved by the US FDA and the EMA for the treatment of non-24-hour sleep–wake disorder (non-24) and by the US FDA for the treatment of nighttime sleep disturbances in Smith–Magenis syndrome (SMS). The SET and RESET studies that supported the approval of tasimelteon for non-24 demonstrated tasimelteon’s circadian entraining properties and its ability to improve sleep parameters [19].

Given the suspected circadian nature underlying the sleep disruption in SMS, we hypothesized that tasimelteon could ameliorate this disruption and would lead to improved sleep in individuals with SMS.

## MATERIALS AND METHODS

### Study design

**Fig. 1:**
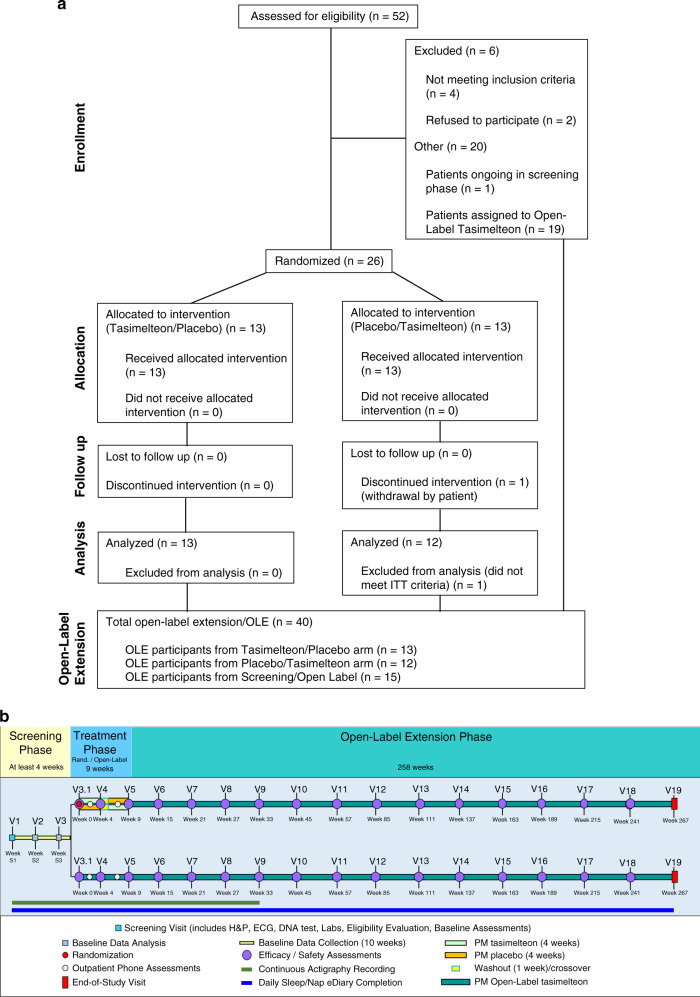
Patient flow diagram and study design. (**a**) CONSORT patient flow diagram. (**b**) The study consisted of a randomized, placebo-controlled, two-phase crossover treatment arm and a parallel open-label treatment arm for patients who did not qualify for the randomization treatment period. Both the randomization and open-label treatment arms were followed by an open-label extension phase.

This study was a placebo-controlled, double-blind, randomized, two-period crossover study followed by an optional open-label extension phase to evaluate the safety and efficacy of tasimelteon in improving the sleep disorder in SMS. As shown in Fig. 1, the study consisted of three phases: a screening phase, a treatment phase, and an open-label extension phase for those who wished to continue treatment. During the treatment phase, patients who did not meet criteria for randomization into the crossover portion were given the option to enroll into open-label. During the crossover portion of the treatment phase, both study patients and the medical professionals interacting with patients were unaware of treatment group assignment. Because SMS is a relatively uncommon syndrome, the population eligible to enroll into this study was limited. A crossover design was chosen to accommodate the small population size available and to increase statistical power.

To be eligible for the study, candidates must have had a confirmed clinical and genetic diagnosis of SMS with chromosomal microarray or targeted sequencing (see Table 1), between the ages of 3 and 65 years of age and had a recent history of sleep disturbances. Patients were also required to have a caregiver able to complete outpatient assessments and to be willing and able to comply with study requirements.

**Table 1:** Smith–Magenis syndrome associated chromosome 17 aberrations.

SUBID	Change	Chromosome	Min interval (hg19)	Min size (Mb)	Number of probes	Max interval* (hg19)	Max size (Mb)
1	LOSS	17p11.2	16892401–20120102	3.228	769	16875775–20130147	3.254
2	LOSS	17p11.2	16843453–20217777	3.374	852	16843269–20702553	3.859
3	LOSS	17p11.2	16843453–19629885	2.786	728	16843269–19664751	2.821
4	LOSS	17p12p11.2	12083553–17900832	5.817	727	12033660–17918519	**5.885**
5	LOSS	17p11.2	16843453–20217777	3.374	838	16843269–20702553	3.859
6	LOSS	17p11.2	16851847–20217777	3.366	829	16845691–20702553	3.857
7	LOSS	17p11.2	17129278–20045186	2.916	674	17127698–20047848	2.920
8	LOSS	17p11.2	16842163–20217777	3.376	843	16551197–20702553	4.151
9	LOSS	17p12p11.2	15784013–20217777	4.434	954	15687411–20702553	5.015
10	LOSS	17p11.2	16842067–20217777	3.376	861	16554197–20702553	4.151
11	LOSS	17p11.2	16842163–20217777	3.376	843	16551197–20702553	4.151
12	LOSS	17p11.2	16842347–20217777	3.375	842	16842215–20702553	3.860
13	LOSS	17p11.2	17084215–19775060	2.691	692	17049726–19790451	2.741
14	—	NP_109590.3:p.(Arg960Ter) (NM_030665.3:c.2878C>T) pathogenic variant					
15	LOSS	17p11.2	17395274–20071903	2.677	592	17251082–20078853	2.828
16	LOSS	17p12p11.2	15176391–21301295	6.125	1023	15168733–21307993	**6.139**
17	LOSS	17p11.2	16842163–20217777	3.376	843	16551197–20702553	4.151
18	LOSS	17p11.2	16842163–20217777	3.376	843	16551197–20702553	4.151
19	LOSS	17p11.2	16842163–20217777	3.376	843	16551197–20702553	4.151
20	LOSS	17p12p11.2	15784013–20217777	4.434	954	15687411–20702553	5.015
21	LOSS	17p11.2	16842347–20217777	3.375	842	16842215–20702553	3.860
22	LOSS	17p11.2	16842163–20217777	3.376	843	16551197–20702553	4.151
23	LOSS	17p11.2	16851847–20217777	3.366	833	16845691–20702553	3.857
24	LOSS	17p11.2	16842163–20217777	3.376	843	16551197–20702553	4.151
25	LOSS	17p11.2	16842163–20217777	3.376	843	16551197–20702553	4.151
26	LOSS	17p11.2	16843360–20217777	3.374	839	16843269–20702553	3.859
27	LOSS	17p11.2	17084215–18267532	1.183	553	17049726–18531458	1.482
28	LOSS	17p11.2	16851847–20217777	3.366	833	16845691–20702553	3.857
29	LOSS	17p11.2	16842163–20217777	3.376	843	16551197–20702553	4.151
30	LOSS	17p11.2	17010338–18012295	1.002	271	17007016–18012422	1.005
31	LOSS	17p11.2	16842163–20217777	3.376	843	16551197–20702553	4.151
32	LOSS	17p11.2	16842347–20217777	3.375	842	16842215–20702553	3.860
33	LOSS	17p12p11.2	15784013–20217777	4.434	343	15687411–20667129	4.980
34	LOSS	17p11.2	16842163–20217777	3.376	843	16551197–20702553	4.151
35	LOSS	17p11.2	16851847–20217777	3.366	833	16845691–20702553	3.857
36	LOSS	17p11.2	16842163–20217777	3.376	843	16551197–20702553	4.151
37	LOSS	17p12p11.2	15784013–18267532	2.484	708	15687411–18289210	2.602
38	LOSS	17p11.2	16842347–20217777	3.375	842	16842215–20702553	3.860

Those who fulfilled these criteria entered the screening phase. Patients who met all initial eligibility criteria at screening began washing out of prohibited medications. Those unwilling or unable to follow the medication restrictions including the washout from use of a prohibited medication were excluded. Prohibited concomitant medications included any medication known to cause sedation or stimulation, dietary supplements and other preparations containing melatonin, and melatonin agonists. Candidates who demonstrated fragmented sleeping and impaired sleep quality during the screening phase were eligible to enter the study.

The study was conducted at four sites in the United States. To reduce the burden on patients and their families, for most visits, patients were permitted to either report to the site closest to them or have a trained nurse conduct the visit in the patient’s home.

During the crossover portion of the study, patients were randomly assigned to one of two treatment sequences. Those assigned to treatment sequence A were dosed with tasimelteon for four weeks, followed by a washout period of one week. Following the washout period, those in treatment sequence A received four weeks of treatment with placebo. Those assigned to treatment sequence B received placebo for four weeks, followed by a washout period of one week. Following the washout period, those in treatment sequence B received four weeks of treatment with tasimelteon.

### Treatment

Two formulations of tasimelteon were used in this study. A capsule (20 mg) was provided for adult patients and an oral suspension administered by 3 ml or 5 ml syringe (4 mg/ml) was provided for pediatric patients. A weight appropriate dosing schedule was developed for the oral suspension in a previous study and administered as ≤28 kg: 0.7 mg/kg or >28 kg: 20 mg. Treatment was administered as a capsule or oral suspension once daily, one hour before target bedtime (±30 minutes). Placebo and tasimelteon were indistinguishable regardless of formulation.

### Statistical analysis

The primary endpoints were the average of 50% worst daily diary sleep quality (DDSQ50) (change from baseline) and the average of the 50% worst daily diary total sleep time (DDTST50). These primary endpoints looked at the worst half of nights for sleep quality (DDSQ50) and total sleep time (DDTST50). These data were collected through a daily diary that the patients’ caregivers completed on provided forms. These endpoints were developed through a preliminary, observational study in which the sleep patterns of SMS patients were observed. During the observational study it was observed that patients had highly variable sleep patterns with nights of good and poor quality sleep and of long and short total sleep time. Thus, to better quantify the effect of tasimelteon in this population, the DDSQ50 and DDTST50 were chosen as primary endpoints. The average of 50% worst nighttime sleep quality (DDSQ50) was calculated as the average of the first half of data, sorted from worst to best, if the total number of data points was even, or the first half +1 of data if the total number of data points was odd. For DDTST50 the same approach was utilized. These endpoints were analyzed in the intent-to-treat (ITT) population by comparing the effect of tasimelteon and placebo. *P* values were calculated using a mixed effects model that included the fixed, categorical effects of treatment, period, and sequence of treatment.

Secondary endpoints that were analyzed included the average daily diary sleep quality (DDSQ), daily diary total sleep time (DDTST), 50% worst daily number of nighttime awakenings, 50% worst latency to sleep, daily number of nighttime awakenings, daily total amount of nighttime sleep, and latency to sleep, all by post sleep questionnaire (PSQ) and actigraphy. Additional secondary endpoints included the average 50% worst daily length of nighttime awakenings, 50% worst daily total amount of nighttime sleep, and daily length of nighttime awakenings measured by actigraphy. Finally, changes in clinical impression were determined as Clinical Global Impression Severity and Change (CGI-S, CGI-C) at each visit, and mixed model analysis was used to analyze the treatment differences using the Aberrant Behavior Checklist (ABC) [21, 22].

Data from the open-label treatment arm and open-label extension are also presented below. The same measures were collected as in the randomization arm and are used to provide additional insight into the persistence of efficacy of tasimelteon.

### Clinical genetic testing

Confirmation of Smith–Magenis diagnosis was determined for each participant by clinical chromosomal microarray assay (CMA) or targeted RAI1 gene sequencing. Participants had a confirmed heterozygous deletion (of chromosome 17p11.2) inclusive of *RAI1* or an *RAI1* pathogenic variant resulting in haploinsufficiency (Table 1). CMA identified a copy-number loss of chromosome band encompassing the RAI1 gene in the Smith–Magenis, as depicted in Table 1. Of the 38 patients, 37 patients had hemizygosity of *RAI1* due to 17p11.2, while one patient had a *RAI1* variant (*RAI1* stop-gain). Moreover two patients' deletions were larger in size and encompassed deletion of CMT1A (Table 1 bold).

## RESULTS

Potential patients were identified from SMS registries operated by Vanda; advocacy groups including PRISMS, Inc.; and through phone and web-based patient outreach campaigns. Patient recruitment for the crossover portion of the study began in 2015 and lasted for approximately 3 years. Figure 1a shows the flow diagram of patients for the study, with 52 people screened as of the data lock point on 3 December 2018 and 46 people completing the screening phase. A total of 26 people were enrolled to the treatment phase of the study, 25 (96.2%) who were randomized completed the treatment phase, and only 1 person (3.8%) withdrew from the treatment phase. Following completion of the treatment phase, randomized patients were offered enrollment into the open-label extension (OLE) phase; however, 19 of the 52 screened study patients (36.5%) entered directly into the OLE phase. Five patients were initially assigned to the open-label phase and then subsequently rescreened into the randomization phase (an additional subgroup analysis excluding these patients is provided below). Supplementary Table 1 summarizes the demographic characteristics for all randomized patients. Overall, demographic and other baseline characteristics were evenly distributed between both treatment arms.

Results from the ITT population were analyzed for all primary and secondary efficacy endpoints. Both primary endpoints, average of 50% worst DDSQ (DDSQ50) and average of 50% worst DDTST (DDTST50), favored tasimelteon over placebo, with DDSQ50 demonstrating a statistically significant improvement over placebo (Table 2). Treatment with tasimelteon showed a difference of 0.4 increase in average sleep quality on the worst 50% of nights (tasimelteon = 2.8, placebo = 2.4, *p* = 0.0139). Total sleep time on the worst 50% of nights resulted in a difference of 18.5 minutes increase for tasimelteon compared to placebo (tasimelteon = 419.3 minutes, placebo = 400.9 minutes, *p* = 0.0556).

**Table 2:** Primary efficacy endpoint analysis.

Primary efficacy endpoint	Placebo (*N* = 25)	Tasimelteon (*N* = 25)	Difference	95% CI	*p* value
Average of 50% worst DDSQ	0.3	0.7	0.4	(0.1, 0.7)	0.0139
Average of 50% worst DDTST—hours (minutes)	0.3 (17.6)	0.6 (36.1)	0.3 (18.5)	(0.0, 0.6)	0.0556

Above values represent change from baseline.

*CI* confidence interval, *DDSQ* average daily diary sleep quality, *DDTST* average daily diary total sleep time.

Secondary endpoints showed both subjective and objective evidence of tasimelteon treatment improving sleep related symptoms. These results complemented and extended the conclusions from the primary endpoints showing improvement in overall sleep quality (tasimelteon = 0.6, placebo = 0.2, *p* = 0.0155) and total sleep time as determined by diary (tasimelteon = 40.9, placebo = 19.8, *p* = 0.0134). Further, actigraphy-based measurement of total sleep time also showed improvement (tasimelteon = 20.2, placebo = 1.9, *p* = 0.0218). Secondary efficacy endpoints are summarized in Table 3.

**Table 3:** Secondary efficacy endpoint analysis.

	Parameter	Placebo	Tasimelteon	Difference	95% CI	*p* value
PSQ	Average of 50% worst daily number of nighttime awakenings	−0.1	−0.3	−0.3	(−0.6, 0.1)	0.1157
	Average of 50% worst latency to sleep (minutes)	−1.4	−6.4	−5.0	(−11.8, 1.8)	0.1393
	Average of daily nighttime sleep quality	0.2	0.6	0.3	(0.1, 0.6)	0.0155
	Average of daily number of nighttime awakenings	0.0	−0.2	−0.3	(−0.5, 0.0)	0.0804
	Average of daily total amount of nighttime sleep—hours (minutes)	0.3 (19.8)	0.7 (40.9)	0.4 (21.1)	(0.1, 0.6)	0.0134
	Average of latency to sleep (minutes)	−0.1	−3.2	−3.2	(−7.9, 1.6)	0.1830
Actigraphy	Average of 50% worst daily length of nighttime awakenings (minutes)	1.7	−3.4	−5.1	(−13.3, 3.1)	0.2017
	Average of 50% worst daily number of nighttime awakenings	−0.3	0.6	0.9	(−0.5, 2.4)	0.1945
	Average of 50% worst daily total amount of nighttime sleep—hours (minutes)	0.0 (2.6)	0.4 (22.4)	0.3 (19.8)	(0.0, 0.6)	0.0308
	Average of 50% worst latency to sleep (minutes)	−0.7	0.1	0.8	(−2.2, 3.7)	0.5920
	Average of daily length of nighttime awakenings (minutes)	2.4	−2.3	−4.7	(−10.3, 0.9)	0.0964
	Average of daily number of nighttime awakenings	0.1	0.5	0.4	(−1.0, 1.8)	0.5851
	Average of daily total amount of nighttime sleep—hours (minutes)	0.0 (1.9)	0.3 (20.2)	0.3 (18.2)	(0.1, 0.6)	0.0218
	Average of latency to sleep (minutes)	0.1	0.0	−0.1	(−1.8, 1.6)	0.9297
CGI	Clinical Global Impression—Severity (CGI-S)^a^	−0.2	−0.6	−0.4	(−0.9, 0.1)	0.0914
	Clinical Global Impression—Change (CGI-C)^a^	3.6	3.0	−0.6	(−1.3, 0.1)	0.0885

Values above represent change from baseline, except where noted.

*CI* confidence interval, *PSQ* post sleep questionnaire.

^a^Postbaseline.

Subgroup analysis was performed after removing the five patients who were randomized after having enrolled in open-label treatment (Table 4). The results of this subgroup analysis were similar to the full ITT. Both primary endpoints (DDSQ50 and DDTST50) demonstrated statistical and clinical significance, as did the overall average (DDSQ and DDTST).

**Table 4:** Subgroup analysis: intention-to-treat (ITT) excluding five re-enrolled patients.

	Placebo (*N* = 20)	Tasimelteon (*N* = 20)	Difference	*p* value
DDSQ50	0.1	0.6	0.5	0.0056
Total DDSQ	0.1	0.5	0.4	0.0053
DDTST50 (minutes)	6.3	29.7	23.4	0.0083
Total DDTST (minutes)	6.2	33.0	26.8	0.0028

*DDSQ* average daily diary sleep quality, *DDSQ50* average 50% worst daily diary sleep quality, *DDTST* average daily diary total sleep time, *DDTST50* average 50% worst daily diary total sleep time.

The open-label extension study showed consistent improvement in sleep with tasimelteon treatment in DDSQ50, DDSQ, DDTST50, and DDTST (Supplementary Table 2). The magnitude of improvement in these sleep related symptoms was equivalent across treatment phases, providing further evidence of the treatment effect. Further, objective measures of sleep, i.e., actigraphy, showed that total sleep time was similar across the double-blind and open-label periods of treatment. Interestingly, a larger improvement in behavioral symptoms was observed in patients treated for longer than 90 days as measured by the ABC (Supplementary Table 25).

Safety analyses were conducted for both the treatment phase and the open-label extension. Overall, tasimelteon was safe and well tolerated. For the treatment phase, 7 (26.9%) patients experienced a treatment emergent adverse events (TEAE) while on placebo, and 6 (24.0%) patients experienced a TEAE while on blinded tasimelteon. Ten (52.6%) patients reported a TEAE while on open-label tasimelteon. One (4.0%) patient on blinded tasimelteon and one (5.3%) patient on open-label tasimelteon each experienced a TEAE that led to a temporary disruption of the study drug; no patients experienced any TEAEs that led to study drug being withdrawn. There were no serious adverse events or deaths during the treatment phase. There were no clinically meaningful changes in mean chemistry, hematology, or urinalysis values over time. In general, the adverse events experienced were common for the study population and similar between placebo and tasimelteon for the treatment phase.

## DISCUSSION

Tasimelteon provided a significant improvement in sleep in patients with SMS treated for 4 weeks in a double-blind, placebo-controlled, crossover study. This improvement was observed in the primary endpoint of 50% worst nights of sleep quality (DDSQ50) (difference = 0.4, *p* = 0.0139). The second primary endpoint did not reach statistical significance but showed an average improvement of 18.5 minutes of sleep (*p* value = 0.0556) on the worst 50% of nights (DDTST50). In addition to the improvement demonstrated on the primary endpoints, tasimelteon improved additional subjective and objective measures that characterize both the quality and length of sleep. This is particularly noteworthy given that sleep disturbances in those with SMS have been difficult to improve; in fact, in a study assessing the use of prolonged-release melatonin to improve insomnia in children with an autism spectrum disorder and/or neurodevelopmental disorder, results showed a greater improvement in Total Sleep Time (TST) when SMS patients were excluded from analysis [23].

In addition to the effects observed in the crossover portion of the study (4 weeks of treatment), there was an OLE phase into which patients from the randomization and open-label treatment periods could enter. During the OLE phase, tasimelteon continued to show improved sleep by both objective and subjective measures. Interestingly, while the ABC checklist that quantifies behavioral symptoms in SMS did not show statistically significant improvement in the 4-week randomization phase, a large improvement emerged in the OLE phase when the length of treatment was extended for 3 months or longer. This suggests that sleep improvements take additional time to engender behavioral improvements and is consistent with previous findings [23].

One limitation of this study is the relatively small sample size due to the rarity of the disorder and the difficulty of patients to participate in a controlled study, which limits the types of analyses and evaluations of the efficacy of the drug. Another limitation of the study is the duration of the washout period of one week; however, the washout was limited to one week to reduce the amount of time patients were without treatment. Due to logistics, and difficulty bringing in patients for the serum melatonin measurements, melatonin was not measured in this current study. That is one limitation that could be addressed in future studies either via serum melatonin measurement or via salivary dim light melatonin onset (DLMO) tests.

The results of this study are consistent with the putative mechanism of action of tasimelteon as a circadian regulator in non-24 and jet-lag disorder [18, 19]. The underlying genetic etiology of SMS, haploinsufficiency of *RAI1* due to chromosome 17p11,2 deletion or pathogenic variant in *RAI1*, causes circadian dysfunction in patients, leading to a myriad of symptoms, most critically, sleep disruption. In this study, we provide evidence that tasimelteon can safely provide persistent improvement of the sleep disturbances associated with SMS and may ultimately improve the quality of life for patients and their families. Additional longitudinal real world studies should be conducted to evaluate the benefits of tasimelteon in improving both sleep and behavioral symptoms of patients with SMS.

## Supplementary information

Supplemental material.
