# Supplementary material for: Tasimelteon safely and effectively improves sleep in Smith–Magenis syndrome: a double-blind randomized trial followed by an open-label extension
Source: Genet Med. 2021 Jul 27;23(12):2426–32. doi: 10.1038/s41436-021-01282-y (PMC8629754; doi:10.1038/s41436-021-01282-y)
Supplement: Supplementary file 1 — Supplemental material. [file 41436_2021_1282_MOESM1_ESM.docx]

**Supplementary Table 1. Demographics of Randomized Patients**

| **Characteristic**  **Statistic or Category** | **Sequence A**  **(Tasimelteon/**  **Placebo)**  **(N=13)** | **Sequence B**  **(Placebo/ Tasimelteon)**  **(N=13)** | **Total**  **(N=26)** |
| --- | --- | --- | --- |
| Sex (n, %) | | | |
| Male | 5 (38.5) | 6 (46.2) | 11 (42.3) |
| Female | 8 (61.5) | 7 (53.8) | 15 (57.7) |
| Age (years) | | | |
| n | 13 | 13 | 26 |
| Mean (SD) | 14.7 (8.04) | 20.1 (10.30) | 17.4 (9.46) |
| Age group (n, %) | | | |
| Pediatric (3 to <16 years) | 6 (46.2) | 5 (38.5) | 11 (42.3) |
| Adult (16 to 65 years) | 7 (53.8) | 8 (61.5) | 15 (57.7) |
| Race (n, %) | | | |
| Asian | 1 (7.7) | 0 (0.0) | 1 (3.8) |
| White | 12 (92.3) | 13 (100.0) | 25 (96.2) |
| Ethnic group (n, %) | | | |
| Hispanic or Latino | 1 (7.7) | 0 (0.0) | 1 (3.8) |
| Not Hispanic or Latino | 12 (92.3) | 13 (100.0) | 25 (96.2) |
| Height (cm) | | | |
| n | 13 | 13 | 26 |
| Mean (SD) | 138.7 (25.51) | 146.8 (23.39) | 142.8 (24.34) |
| Weight (kg) | | | |
| n | 13 | 13 | 26 |
| Mean (SD) | 55.35 (27.058) | 54.50 (25.509) | 54.92 (25.767) |
| Body mass index (kg/m^2^) | | | |
| n | 13 | 13 | 26 |
| Mean (SD) | 26.90 (8.909) | 23.55 (6.282) | 25.22 (7.743) |

*^SD=standard deviation; cm=centimeter; kg=kilogram; m=meters^*

**Supplementary Table 2. Endpoints in Patients with ≥ 90 Days Exposure to Tasimelteon**

|  | **Randomization Phase – 4 Week Treatment**  **N = 25** | **Open-label Extension (all patients with ≥ 90 days of exposure)**  **N = 35** |
| --- | --- | --- |
| DDSQ50, mean (SD) | 0.7 (0.83) | 0.7 (0.9) |
| DDSQ, mean (SD) | 0.5 (0.69) | 0. 6 (0.8) |
| DDTST50 in minutes, mean (SD) | 36 (56.05) | 51.8 (55.21) |
| DDTST in minutes, mean (SD) | 39.8 (51) | 49.2 (50.02) |
| ACTITST50 in minutes, mean (SD) | 26.9 (43.48) | 16.0 (35.67) |
| ACTITST in minutes, mean (SD) | 18.7 (39.89) | 18.6 (28.42) |
| ABC Total, mean (SD) | -6.3 (16.28) | -16.6 (14.71) |

*^DDSQ50=Average 50% worst daily diary sleep quality; DDSQ=Average daily diary sleep quality; DDTST50=Average 50% worst daily diary total sleep time; DDTST=Average daily diary total sleep time; ACTITST50=Average 50% worst total sleep time measured by actigraphy; ACTITST= Average total sleep time measured by actigraphy; ABC=Aberrant Behavior Checklist^*
